# Dosimetric Comparisons between Proton Beam Therapy and Modern Photon Radiation Techniques for Stage I Non-Small Cell Lung Cancer According to Tumor Location

**DOI:** 10.3390/cancers13246356

**Published:** 2021-12-17

**Authors:** Unurjargal Bayasgalan, Sung Ho Moon, Tae Hyun Kim, Tae Yoon Kim, Seung Hyun Lee, Yang-Gun Suh

**Affiliations:** 1 Proton Therapy Center, Research Institute and Hospital, National Cancer Center, Goyang 10408, Korea; bayasgalan@ncc.re.kr (U.B.); shmoon@ncc.re.kr (S.H.M.); k2onco@ncc.re.kr (T.H.K.); tykim@ncc.re.kr (T.Y.K.); casper@ncc.re.kr (S.H.L.); 2 Department of Radiation Oncology, National Cancer Center, Ulaanbaatar 13370, Mongolia

**Keywords:** proton beam therapy, non-small cell lung cancer, mean lung dose, mean heart dose, dosimetric comparison

## Abstract

**Simple Summary:**

Stereotactic ablative radiotherapy (SABR) is a well-established technique used to treat stage I non-small cell lung cancer (NSCLC). Proton beam therapy (PBT) offers dosimetric advantages over photon SABR techniques by reducing doses to normal organs. Hence, it is believed that PBT is helpful for patients with tumors located centrally in stage I NSCLC. However, the benefits of PBT for other locations, such as peripherally located tumors, have not been well-described. We investigated dosimetric benefits for PBT over modern photon radiation techniques for stage I NSCLC according to tumor locations. A total of 42 patients’ (including tumors that were central (11), peripheral (nine), and close to the chest wall (22)) PBT plans were compared with those of modern photon radiation techniques. In all locations, PBT significantly reduced radiation exposure to the lung and heart. To reduce potential lung and heart toxicities, PBT is ideal, even in the peripherally located stage I NSCLC.

**Abstract:**

Herein, we investigated the dosimetric benefits for proton beam therapy (PBT) over modern photon radiation techniques according to tumor location (central, peripheral, and close to the chest wall) for stage I non-small cell lung cancer (NSCLC) patients. A total of 42 patients with stage I NSCLC were treated with PBT with a total dose of 50–70 Gy in four or 10 fractions considering the risk of treatment-related toxicities. Simulation plans for three-dimensional conformal radiation therapy (3D-CRT), static-field intensity-modulated radiotherapy (IMRT), and volumetric-modulated arc therapy (VMAT) were retrospectively generated using the same treatment volumes as implemented in the PBT plans for these patients. Dosimetric improvements were observed with PBT as compared with all the photon-based radiation techniques with regards to the mean lung dose, lung V5 and V10, mean heart dose, and heart V5 and V10 in all locations. Moreover, lower radiation exposure to the chest wall was observed within PBT for peripherally located and close to the chest wall tumors. All radiotherapy modalities achieved clinically satisfactory treatment plans in the current study. Notably, the usage of PBT resulted in significant dosimetric improvements in the lung and heart over photon-based techniques at all tumor locations, including the periphery, for stage I NSCLC.

## 1. Introduction

Patients with lung cancer show poor overall survival, and lung cancer has been a leading cause of cancer-related mortality worldwide for many years [1]. However, successful treatment is more likely to be achieved when lung cancer is diagnosed early [2,3]. Stereotactic ablative radiotherapy (SABR) is a well-established technique and is a standard of care for medically inoperable patients or for those who refuse surgery for stage I non-small cell lung cancer (NSCLC) [4]. A slightly protracted hypofractionated schedule may be considered for centrally located tumors or for tumors abutted to the chest wall and brachial plexus in order to avoid severe toxicity associated with high fractionation doses [5,6]. However, these intensive regimens provide a higher tumor control rate and improved survival outcomes as compared to conventionally fractionated radiation therapy [7,8,9].

Although three-dimensional conformal radiotherapy (3D-CRT) remains a popular modality for delivering SABR (among alternatives within photon-based radiotherapy), fixed-beam intensity-modulated radiation therapy (IMRT) [10] and volumetric-modulated arc therapy (VMAT) [11,12] based on SABR are gaining precedence because of their ability to improve dose conformality and treatment efficacy, as well as their favorable dosimetry properties. Previous studies have shown that proton beam therapy (PBT) offers dosimetric advantages over these photon-based SABR techniques by reducing doses to critical normal organs and structures, including the lung, heart, esophagus, and spinal cord [13,14]. Hence, the Particle Therapy Co-Operative Group (PTCOG) addressed the advantages of PBT for early-stage NSCLC in a comprehensive report; among other findings, this task group concluded that patients with larger tumors or tumors located either centrally or close to the brachial plexus may benefit more strongly from treatment with PBT [15]. However, the benefits of PBT for stage I tumors in other locations, such as peripherally located tumors, are still being investigated overall and with respect to technological improvements.

Thus, the aim of this dosimetric study was to explore an extensive dosimetric comparison between PBT and photon-based simulation radiotherapy techniques, including 3D-CRT, IMRT, and VMAT, with regard to tumor locations for stage I NSCLC patients treated with SABR and hypofractionated radiotherapy.

## 2. Materials and Methods

### 2.1. Patients

Our previous study investigated treatment outcomes for 42 patients with stage I (T < 5 cm, N0) NSCLC (according to the 7th edition of the American Joint Committee on Cancer [AJCC] TNM classification) who were treated with PBT between 2016 and 2019 [16]. In the current study, the dosimetric parameters for these patients’ PBT plans were compared with simulation 3D-CRT, IMRT, and VMAT plans based on the tumor location (central, peripheral, and close to the chest wall). Briefly, the median age of the enrolled participants was 78 years (range, 58–92 years), the majority of patients were male (*n* = 27, 64%), and 50% of the patients’ tumor histology were adenocarcinoma (*n* = 21). Patients were treated with a prescribed dose of 50–70 CGE in four or 10 fractions (Table 1). This study was approved by the Institutional Review Board of National Cancer Center in the Republic of Korea (2020-0076). The data for this study, though not available in a public repository, will be made available to other researchers upon reasonable request.

### 2.2. Radiotherapy Simulation and PBT Planning

All the patients underwent a contrast-enhanced four-dimensional (4D) CT-based treatment simulation with a 3-mm-slice thickness in a supine position on a round couch. Respiratory motion was accounted for using a real-time position management (RPM) system (Varian Medical Systems, Palo Alto, CA, USA). All patients were treated with respiratory-gated PBT. Gating windows were defined as ranging from 40 to 60%. Target volumes for the gross tumor volume (GTV), the internal GTV, the planning target volume (PTV), and the volumes of organs at risk (OARs) were delineated within the gating windows of 4D-CT.

**Figure 1:**
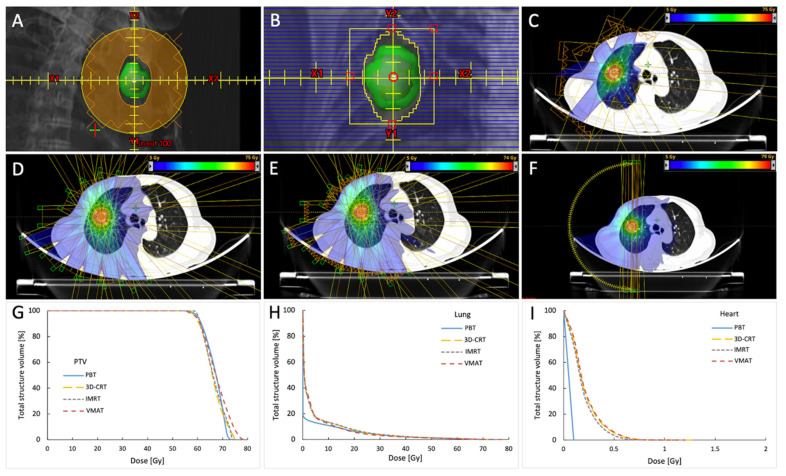
Examples of block and MLC settings, each treatment plan’s dose distributions and beam arrangements, and dose–volume histogram for the PTV, lung, and heart for the peripherally located tumor treated with 60 Gy in 4 fractions. The GTV is showed in red, iGTV in magenta, and PTV in green contours. Blocks were made with 1–3-mm lateral margins and 7 to 8-mm superior–inferior margins with respect to the PTV (shown in the green structure; the reddish structure inside is iGTV) (**A**). MLC was set manually for 3D-CRT, and no additional margin was added at the x-axis edges, and 0.7 cm was added to the y-axes of the MLC (**B**). Examples of dose distributions and beam arrangements for the PBT (**C**), 3D-CRT (**D**), IMRT (**E**), and VMAT (**F**) plans. Corresponding dose–volume histogram for PTV (**G**), the lung (**H**), and the heart (**I**). MLC, multileaf collimator; PTV, planning target volume; PBT, proton beam therapy; 3D-CRT, three-dimensional conformal radiotherapy; IMRT, intensity-modulated radiotherapy; VMAT, volumetric-modulated arc therapy.

The proton beam treatment plans were generated using an Eclipse treatment planning system (TPS) with a proton convolution superposition algorithm implemented for the calculations (Varian Medical Systems). Protons were delivered in 3 to 4 coplanar beams using the system’s passive scattering mode with a proton mass energy equivalent of 230 MeV. Plans were prescribed for the PTV and the field-specific PTV and were normalized such that at least 95% of the PTV was to be encompassed with >99% of the prescription dose. Customized compensators and blocks with 1–3-mm lateral margins and 7 to 8-mm superior–inferior margins with respect to the PTV were made for each patient (Figure 1A).

### 2.3. Photon-Based Simulation Treatment Planning

All photon-based simulation plans used the same volumes for the original treatment planning as PBT; these plans were generated via an Eclipse TPS on a Trilogy Linac (Varian Medical Systems) consisting of high-definition (HD) 120 multileaf collimators (MLC); 6 MV flattening filter-free (FFF, 1400 MU/min) beams were implemented. The central 64 leaves of the HD120 MLC have a leaf width of 2.5 mm, and the peripheral 56 leaves have a 5-mm width at a source axis distance of 100 cm. Although this results in a maximal field height of 22 cm, HD120 MLC provides a more optimal dose conformality and is thus suitable for precisely delivering an ablative dose. Eleven coplanar beams were manually selected in both 3D-CRT and static-field IMRT plans for SABR (4 fractions); nine coplanar beams were used for hypofractionated radiotherapy (10 fractions); and selected beams avoided the contralateral lung, spinal cord, and the major portion of the heart. The MLC was set manually for 3D-CRT (i.e., no additional margin was added at the x-axis edges, and 0.7 cm was added to the y-axes of the MLC (Figure 1B); the leaf edge–contour meet point was set inside for SABR and was set at the middle for the hypofractionated cases), and the beam shapes were optimized using MLC for IMRT with a minimum of 350 iterations (at which point, the cost function converged). The volumetric-modulated arc therapy plans used two half-rotation coplanar arcs to spare the contralateral lung with a cumulative arc length of approximately 340–358°. Collimator angles of 30° and 330° were set manually in order to reduce MLC tongue-and-groove dose leakage throughout the arc rotation. Each plan was validated to ensure that the plans were clinically satisfactory after optimization. If needed, the plans were reoptimized. Dose calculations were performed using an analytic anisotropic algorithm (AAA) for all photon-based plans. Isocenters were set to the geometric center of the PTV. Prescription lines covering the PTV were typically 75–85% of the maximum dose (Dmax), with an accepted range of 60–90%. Hotspots only existed within the PTV for the SABR plans. For the hypofractionated radiotherapy plans, dosimetric criteria mandated that at least 95% of the PTV was covered by >99% of the prescription dose. All PBT plans were generated by board-certified dosimetrists, whereas all simulation photon plans were completed by a specially appointed physician to ensure consistency of the radiation plan.

### 2.4. Plan Evaluation

The plan quality of SABR was assessed using the conformity index (CI) and the gradient index (GI) according to the RTOG lung SABR protocols [17,18]. The hypofractionated radiotherapy plan quality was assessed by verifying the prescription dose coverage to the target volume, along with the Dmax and the minimum dose (Dmin) for the PTV. Furthermore, we evaluated the dose–volume histogram (DVH) of all the treatment plans. To compare plans with different modalities in terms of OARs, the Dmax of the spinal cord, esophagus, great vessels, hilar major vessels, proximal bronchial tree, chest wall, and skin were recorded. Dose–volume parameters were collected for the lung and heart. The definition of OARs and their dose constraints were adapted from previous studies [5,19]. We evaluated absolute doses in order to compare dosimetric parameters between the radiotherapy techniques.

### 2.5. Statistical Analysis

Medians and ranges for descriptive statistics were calculated in order to summarize the participant characteristics with respect to the dosimetric parameters of interest. Statistical differences for the dosimetric parameters were determined using two-tailed Wilcoxon Signed Rank tests for comparisons between nonparametric paired data within each group. *p*-values of <0.05 were considered statistically significant. All statistical analyses were performed using R statistical software (version 4.0.2; R Foundation for Statistical Computing, Vienna, Austria [20]) in RStudio (version 1.3.1073; RStudio, PBC, Boston, MA, USA).

## 3. Results

### 3.1. Target Coverage

With respect to the SABR plans, we found that both IMRT and VMAT provided relatively lower CI and GI values compared with 3D-CRT and PBT (Table S1). The median CI values for PBT, 3D-CRT, IMRT, and VMAT were 1.21, 1.19, 1.02, and 1.04, respectively. The median GI values were 4.49, 4.88, 4.42, and 4.24 for PBT, 3D-CRT, IMRT, and VMAT, respectively.

In the hypofractionated cases, the median Dmax percentages of the prescribed dose to the PTV were 115.5%, 120.4%, 110.0%, and 112.6% for PBT, 3D-CRT, IMRT, and VMAT, respectively (Table S1). The median minimum dosage (Dmin) values for PBT, 3D-CRT, IMRT, and VMAT were 86.8%, 86.8%, 93.3%, and 90.3%, respectively.

### 3.2. Lung

**Figure 2:**
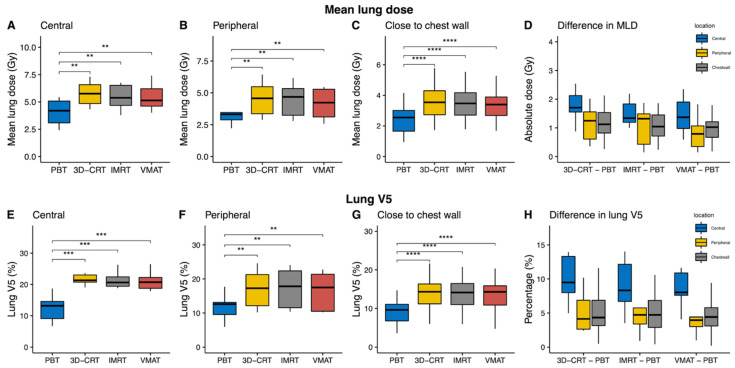
Mean lung dose (MLD) and lung V5 comparisons according to treatment modality and with regard to tumor locations. Dosimetric parameters for proton beam therapy (PBT) were statistically significantly improved as compared with other photon plans at each location. (**A**–**C**) Boxplots of the MLD are shown in units of absolute dose per location. (**D**) Boxplots of median differences in the MLD between photon-based radiotherapy techniques and PBT. (**E**–**G**) Boxplots of the percentage of the lung receiving 5 Gy in each subgroup. (**H**) Median differences in lung V5 between photon-based radiotherapies and PBT. ** *p* ≤ 0.01; *** *p* ≤ 0.001; **** *p* ≤ 0.0001.

In all patients, the lung V5 and V10, as well as the mean lung dose (MLD), were statistically significantly lower in the PBT plan as compared with all the other photon-based plans (all *p* ≤ 0.002; Table S1 and Figure S1). In contrast, the lung V30 and V40 were statistically significantly lower within IMRT and VMAT as compared with PBT; however, the absolute median differences were less than 0.7%. These dosimetric improvements in lung V5 and V10 and in the MLD were also demonstrated within the subgroups, with variations according to tumor location, including central tumors (Table 2), peripheral tumors (Table 3), and tumors close to the chest wall (Table 4). The MLD and lung V5, which varied according to radiotherapy techniques and tumor locations, are shown in Figure 2. The reductions in the MLD and in lung V5 following PBT were largest in the central tumors (Figure 2D,H).

### 3.3. Heart

**Figure 3:**
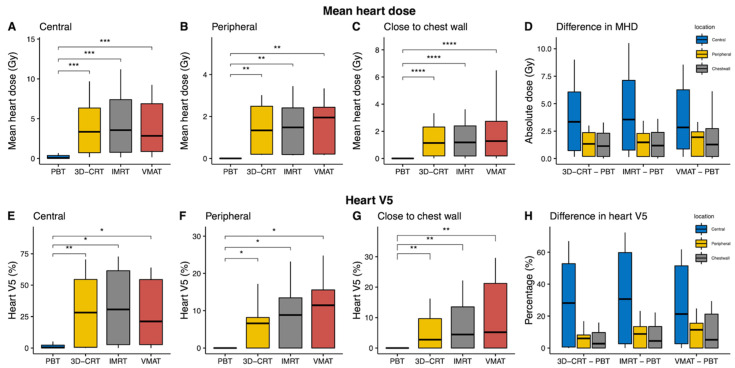
Mean heart dose (MHD) and heart V5 comparisons according to treatment modality and tumor location. We found that proton beam therapy (PBT) provided a statistically significantly reduced MHD and V5 as compared to the other photon-based radiotherapy techniques at each location. (**A**–**C**) Boxplots of the MHD are shown in units of absolute dose per location. (**D**) Boxplots of the median differences in the MHD between photon-based radiotherapy techniques and PBT. (**E**–**G**) Boxplots of the percentage of the heart receiving 5 Gy in each subgroup. (**H**) Median differences in the heart V5 between photon-based radiotherapies and PBT. * *p* ≤ 0.05; ** p ≤ 0.01; *** *p* ≤ 0.001; **** *p* ≤ 0.0001.

The benefits of PBT with regards to heart sparing were similar to those of the lung. Heart V5 and V10 and the mean heart dose (MHD) in the PBT plan were statistically significantly lower as compared with all other photon-based plans in all the patients enrolled in the current study (all *p* ≤ 0.002; Table S1 and Figure S1). The benefits of PBT were consistent in the central tumors (Table 2), peripheral tumors (Table 3), and tumors close to the chest wall (Table 4). Improvements with respect to the MHD and heart V5 were the largest in the central tumors (Figure 3D,H).

### 3.4. Other OARs

In all patients, the Dmax and the highest dose delivered to 1 cc (D_1cc_) of the proximal bronchial tree were statistically significantly lower in PBT as compared with photon-based radiotherapy (Table S1). However, in the subgroup analyses according to tumor location, these differences did not reach statistical significance (Table 2, Table 3 and Table 4). For the great vessels and hilar major vessels, PBT did not show dosimetric benefits as compared with photon-based radiotherapy.

The Dmax for the spinal cord and the Dmax and mean dose (Dmean) for the esophagus were statistically significantly lower in PBT as compared with all the other photon-based radiotherapies (Table S1); these statistic significances were also shown in the subgroup analyses in the central tumors, peripheral tumors, and tumors close to the chest wall (Table 2, Table 3 and Table 4). With respect to the chest wall, PBT showed statistically significant dose improvements for D_30cc_ in peripheral tumors (Table 3) and for Dmax in tumors close to the chest wall as compared with all the other photon-based techniques (Table 4). With respect to skin dosages, reductions of the Dmax via PBT were observed in peripheral tumors (Table 3) and tumors close to chest wall (Table 4).

## 4. Discussion

All four modalities evaluated in the current study achieved satisfactory treatment plans using SABR and hypofractionated techniques for stage I NSCLC from the target coverage standpoint with regard to dose conformity and fall-off outside the target. Although the static field IMRT and VMAT techniques demonstrated better conformity and distributions of higher doses as compared with 3D-CRT and PBT, PBT was superior with respect to sparing the lungs and heart at all locations, including central, peripheral, and close to the chest wall locations. 

The RTOG lung SABR protocols [17,18] recommend the use of a minimum of seven beams for photon-based treatment planning. This approximate number of beams are generally used in clinical practice to reduce the treatment time. The number of static beams used for photon plans in the current study was either 9 or 11, whereas three to four coplanar beams are normally used for PBT. All SABR cases with respect to the simulation photon plans and PBT abided by the RTOG protocol recommendations with regards to plan conformality. In addition, isodose lines covering the periphery of the PTV were typically 75–85% of the maximum dose used in photon-based plans for the SABR technique in order to acquire a steeper dose gradient outside the target. Due to these prescription differences in PBT and photon-based radiotherapy techniques, the IMRT and VMAT plans produced more conformal plans compared with 3D-CRT and PBT, as demonstrated by the CI and GI values. Although the CI was within the recommended range, PBT tends to have slightly higher values as compared with IMRT and VMAT in order to compensate for uncertainties; other possible reasons for these discrepancies include a smaller number of beams and differing doses prescribed to the isocenter of the target. Nevertheless, a previous study showed that a slightly higher CI value was not associated with the increased incidence of radiation-induced toxicity [21]. Therefore, the clinical impacts of larger CI of PBT could be negligible in the current study.

Based on the results of the current study, PBT provided a clear advantage among all treatment planning techniques in terms of sparing OARs. The MLD and lung V5, the MHD and heart V5, the Dmax of the spinal cord and skin, and the Dmean and Dmax of the esophagus were all statistically significantly improved with PBT at all locations as compared with all the other simulation photon plans. Lower radiation exposure to the chest wall and proximal bronchial tree were observed with PBT delivered to tumors located close to the chest wall. The parameters of the great vessels and hilar major vessels were comparable among the treatment modalities at any location. 

Reducing the dose exposure to critical normal organs is an important consideration during thoracic irradiation. Previous studies have determined the dosimetric parameters associated with the risk of heart disease [22], reduced overall survival (OS) [23], and the development of radiation-induced toxicity [24,25]. Darby et al. [22] reported that the risk of ischemic heart disease increases proportionally to MHD by 7.4% per Gy; this elevated risk remains for as long as 20 years in breast cancer patients. Furthermore, multivariable analyses for OS within the long-term results of RTOG 0617 [23] demonstrated that heart V5 is statistically significantly associated with OS; their separate trial of the impact of the radiation dose on the heart and its substructures is currently underway. Although SABR offers a steep dose gradient outside the target, we found that low-dose exposure to the heart was statistically significantly larger in photon-based techniques as compared with PBT due to the nature of photon beams. The median differences in the MHD between PBT and 3D-CRT, IMRT, and VMAT were approximately 1.6 Gy, favoring PBT in all 42 cases. While these results remained similar at approximately 1.1–1.9 Gy in the subgroups of patients with tumors in or near the chest wall and in peripheral locations, the median differences for the MHD were approximately 3 Gy in the central location groups (Figure 3).

Previous studies have demonstrated that symptomatic radiation pneumonitis (RP) following SABR in early-stage NSCLC occurs in approximately 10–20% of treated patients [25,26,27]. Lung V20 and MLD were the most-reported dosimetric predictors of symptomatic radiation pneumonitis [21,25,26,27]. Moreover, several studies found that V10 was also a significant factor for symptomatic RP [28,29]. The association between symptomatic RP and other lung volume dose metrics such as V30 and V40 was inconsistent in previously published papers [21,28,29]. In the current study, the PBT provided a statistically significantly lower MLD, as well as statistically significantly lower V5 and V10 within low-dose regions in the lungs as compared to the other photon-based techniques. The absolute median differences in MLD between PBT and the other techniques ranged from 1.0 to 1.25 Gy. The percentage of lung V20 in PBT was statistically significantly lower compared to 3D-CRT, had no statistical difference with IMRT, and was statistically significantly higher than VMAT. Only lung V30 and V40 were lower in the photon-based radiotherapy techniques as compared to PBT in tumors located peripherally or close to the chest wall. This result can be explained by the use of higher beam number than PBT or arc therapy in photon-based radiotherapy. As mentioned earlier, the impact of V30 and V40 on symptomatic RP is uncertain, and further research is warranted. These dose reductions for the heart and lungs seen with PBT at all locations suggest that PBT may be a favorable treatment option for stage I NSCLC, along with other OARs providing dosimetric benefits.

Rib fractures and chest wall pain are some adverse events occurring after SABR for stage I NSCLC [30]. Previous studies have demonstrated that these complications are associated with a radiation dose to the chest wall [31,32]. In the current study, PBT reduced the radiation dose to the chest wall in peripheral tumors, as well as tumors close to the chest wall. Therefore, the benefits of PBT regarding chest wall toxicities in these tumors should be investigated in future studies.

There may be a substantial increase in the entrance dose, as Bragg peaks of proton beams with different energies are spread out to create a uniform, in-depth dose across the target. Therefore, there are concerns about skin toxicities for patients treated with PBT [33]. However, in the current study, skin doses in the photon-based radiotherapies were higher than those observed within PBT for the peripheral tumors and tumors close to the chest wall. A possible explanation for this observation may be related to differing entrance and exit doses of photon beams, which may have overlapped, consequently resulting in an increased skin dose as compared to PBT.

In addition to the substantial strengths of the current investigation, one of the limitations of our study is the possible selection bias due to the retrospective dosimetric analysis. However, this selection bias may be negligible, because our group has treated all patients with stage I NSCLC using PBT since 2016. All patients with stage I NSCLC treated with four or 10 fractions by our group during the study period were included in the current study. In addition, to make reliable static IMRT and VMAT simulation plans, all simulation plans were produced and reviewed by a specially appointed physician and experienced dosimetrists. Additionally, the clinical impacts of these theoretical dosimetric benefits are hard to estimate due to the respiratory organ motion, geometric uncertainties, and range uncertainties of PBT. Thus, the planning and treatment were performed using respiratory gating techniques to decrease the interplay effects by organ motion; IGRT was used to decrease the geometric uncertainties; and three or four beams were used for PBT to reduce the range uncertainties of PBT.

To the best of our knowledge, this is the largest study of patients with stage I NSCLC analyzing the dosimetric advantages of PBT compared with those of modern photon radiation techniques. Moreover, we classified stage I NSCLC into central tumors, peripheral tumors, and tumors close to the chest wall and performed statistical analyses for the paired data. As a result, we could reveal dosimetric benefits with regards to radiation dose reductions in the lungs, heart, chest wall, and skin via PBT based upon the tumor location.

## 5. Conclusions

Our results demonstrated that the usage of PBT, 3D-CRT, IMRT, and VMAT for lung SABR and hypofractionated radiotherapy all presented efficient and reliable methods for achieving clinically satisfactory plans for treating stage I NSCLC. However, PBT had the advantage of providing demonstrable improvements in lung and heart sparing at any tumor location, including the peripheral tumors. The results of our investigation inform future research directions, and, if confirmed, will ultimately inform medical guidelines and optimal clinical decision-making.

## Figures and Tables

**Table 1:** Characteristics of the patients.

Characteristics			No. (%)
Sex (%)			
	Male		27 (64)
	Female		15 (36)
Age (years)			
	Median		78
	Range		58–92
Tumor histological type			
	Adenocarcinoma		21 (50)
	Squamous cell carcinoma		9 (21)
	NOS		2 (5)
	Sarcomatoid		1 (2)
	Unproven		9 (21)
Tumor location			
	Central		11 (26)
		2 cm within proximal bronchial tree	1 (2)
		2 cm within mediastinum	10 (24)
	Peripheral		9 (21)
	Peripheral but 1 cm within chest wall		22 (52)
		Close to brachial plexus	1 (2)
		Close to chest wall	21 (50)
T stage			
	T1a		16 (38)
	T1b		17 (41)
	T2a		9 (21)
Total dose/fractions, (BED_10_ *)			
	60 CGE/4 fx (150)		11
	50 CGE/4 fx (112.5)		22
	70 CGE/10 fx (119)		6
	60 CGE/10 fx (96)		3
PTV (cm^3^)			
	Median		34.55
	Range		9.6–84

* BED10, biologically effective dose using α/β ratio of 10. NOS, not otherwise specified; CGE, cobalt gray equivalent; fx, fraction; PTV, planning target volume; cm^3^, cubic centimeter.

**Table 2:** Summary and comparison of the dosimetric parameters of centrally located tumors (50 CGE/4 fx = 4, 70 CGE/10 fx = 5, and 60 CGE/10 fx = 2).

OARs		PBT		3D-CRT		IMRT		VMAT		*p*-Value		
		Median	Range	Median	Range	Median	Range	Median	Range	PBT vs.3D-CRT	PBT vs.IMRT	PBT vs.VMAT
Total lung (*n* = 11)												
	V5 (%)	13.17	6.7–18.7	21.31	19.0–28.7	20.63	14.5–31.2	20.72	17.9–27.8	**0.001**	**0.001**	**0.001**
	V10 (%)	11.5	6.1–16.2	15.63	10.7–18.9	13.79	9.5–19.2	14.19	9.4–19.8	**0.001**	**0.001**	**0.001**
	V15 (%)	9.95	5.4–12.8	11.66	8.0–15.7	9.58	6.1–16.2	10.14	7.5–14.2	**0.003**	0.067	**0.054**
	V20 (%)	8.1	4.8–10.7	9.01	5.5–12.4	7.93	4.7–12.7	7.56	5.1–12.3	**0.024**	0.898	1
	V30 (%)	5.66	2.9–6.8	5.13	2.9–8.2	5.12	2.6–7.7	4.38	2.4–8.7	0.519	0.966	0.278
	V40 (%)	2.96	1.8–4.5	3.04	1.6–4.2	2.62	1.4–4.2	2.55	1.4–3.85	0.578	0.376	**0.016**
	Mean dose (Gy)	4.2	0.1–5.4	5.75	4.3–7.3	5.38	3.8–6.8	5.14	4.0–7.4	**0.006**	**0.001**	**0.001**
Heart (*n* = 11)												
	V5 (%)	0.39	0–5.3	28.12	0–70.48	30.61	0–72.8	21.22	0–64.0	**0.009**	**0.014**	**0.014**
	V10 (%)	0.14	0–4.0	7.14	0–39.3	7.49	0–51.4	8.65	0–41.5	**0.014**	**0.014**	**0.022**
	V15 (%)	0.06	0–3.1	2.51	0–25.1	2.62	0–29.4	3.58	0–20.5	**0.022**	**0.022**	**0.022**
	V20 (%)	0.02	0–1.99	0.82	0–10.2	0.83	0–14.3	0.96	0–8.7	**0.022**	**0.022**	**0.036**
	V30 (%)	0	0–1.2	0	0–1.9	0	0–3.8	0	0–2.1	1	0.402	0.675
	V40 (%)	0	0–0.5	0	0–0.3	0	0–1.0	0	0–0.6	1	0.583	1
	Mean dose (Gy)	0.07	0–1.13	3.35	0.2–9.7	3.56	0.1–11.2	2.84	0.2–9.2	**0.001**	**0.001**	**0.001**
	Dmax (Gy)	23	0–65.98	25.7	0.8–54.8	29.23	0.7–63.8	28.81	0.8–64.4	0.206	0.147	0.175
Proximal bronchial tree (*n* = 9)												
	Dmax (Gy)	22.1	0.1–69.0	27.14	6.9–77.5	30.06	3.7–69.2	26.29	3.8–71.4	0.164	0.129	0.098
	D1cc (Gy)	12.37	0–65.3	15.71	1.9–61.7	17.33	1.7–60.5	18.45	1.8–62.5	0.164	0.164	0.074
Spinal cord (*n* = 11)												
	Dmax (Gy)	0.01	0–14.1	10.82	3.8–23.3	12.69	5.9–20.2	16.54	6.2–29.6	**0.001**	**0.001**	**0.001**
Esophagus (*n* = 11)												
	Mean dose (Gy)	0.02	0–0.9	4.88	1.6–16.0	5.87	1.6–16.9	6.38	1.7–17.6	**0.001**	**0.001**	**0.001**
	Dmax (Gy)	2.42	0–13.1	16	7.7–29.8	15.67	9.5–36.9	18.5	8.7–26.8	**0.001**	**0.001**	**0.001**
Chest wall (*n* = 7)												
	Dmax (Gy)	56.2	37.2–75.2	59.95	39.4–79.4	58.16	36.8–70.2	54.95	38.0–73.6	0.375	0.937	0.812
	D30cc (Gy)	27.21	18.1–44.0	31.95	22.8–39.6	33.86	22.9–41.6	27.71	21.9–39.7	0.375	0.297	0.469
Skin (*n* = 8)												
	Dmax (Gy)	21.48	9.9–54.8	28.46	15.1–35.9	32.46	18.2–40.6	24.84	17.6–37.8	0.641	0.359	0.496

OARs, organs at risk; PBT, proton beam therapy; 3D-CRT, three-dimensional conformal radiotherapy; IMRT, intensity-modulated radiotherapy; VMAT, volumetric-modulated arc therapy, CGE, cobalt gray equivalent; fx, fractions; V5–40; percentage volume of tissue receiving 5–40 Gy; Dmax, maximum dose; D1cc, the dose delivered to 1 cubic centimeter volume; D30cc, the dose delivered to 30 cubic centimeter volume.

**Table 3:** Summary and comparison of the dosimetric parameters of peripherally located tumors (60 CGE/4 fx = 9).

OARs		PBT		3D-CRT		IMRT		VMAT		*p*-Value		
		Median	Range	Median	Range	Median	Range	Median	Range	PBT vs.3D-CRT	PBT vs.IMRT	PBT vs.VMAT
Total lung (*n* = 9)												
	V5 (%)	12.59	6.0–17.7	17.26	10.2–24.6	17.83	10.2–24.1	17.49	10.2–22.8	**0.004**	**0.004**	**0.004**
	V10 (%)	10.28	5.0–15.0	13.41	7.9–19.9	13.48	8.0–19.2	12.28	6.4–15.6	**0.008**	**0.008**	**0.039**
	V15 (%)	8.37	4.1–12.4	10.32	6.5–16.3	9.87	5.9–15.3	8.54	5.0–11.5	**0.039**	0.055	0.820
	V20 (%)	6.36	3.3–10.9	7.19	4.4–11.3	6.35	4.0–10.5	5.79	3.6–8.6	0.098	0.426	0.098
	V30 (%)	3.88	2.1–8.1	3.6	2.1–6.0	3.46	2.1–5.6	3.17	2.0–5.3	0.098	0.074	**0.004**
	V40 (%)	2.49	1.3–5.7	2.16	1.2–3.4	2.18	1.1–3.2	1.99	1.1–3.1	**0.009**	**0.019**	**0.004**
	Mean dose (Gy)	3.32	1.9–5.2	4.57	2.9–6.4	4.68	2.8–6.2	4.23	2.6–5.5	**0.004**	**0.004**	**0.004**
Heart (*n* = 9)												
	V5 (%)	0	0–0.9	6.62	0–17.2	8.82	0–23.2	11.43	0–24.8	**0.036**	**0.036**	**0.036**
	V10 (%)	0	0–0.4	0.03	0–2.4	0.27	0–4.5	1.1	0–5.0	**0.036**	**0.036**	**0.036**
	V15 (%)	0	0–0.1	0	0	0	0–0.2	0	0–0.6	1	1	0.100
	V20 (%)	0	0	0	0	0	0	0	0–0.1	1	1	0.371
	V30 (%)	0	0	0	0	0	0	0	0	NA	NA	NA
	V40 (%)	0	0	0	0	0	0	0	0	NA	NA	NA
	Mean dose (Gy)	0	0–0.1	1.34	0.2–3.0	1.48	0.1–3.4	1.95	0.2–3.3	**0.009**	**0.004**	**0.004**
	Dmax (Gy)	0.03	0–18.9	10.88	0.6–16.1	12.46	0.6–17.4	13.5	0.5–23.9	0.129	0.129	**0.004**
Proximal bronchial tree (*n* = 5)												
	Dmax (Gy)	10.35	0–41.0	17.24	1.0–48.6	22.09	0.9–37.3	20.79	1.0–35.0	0.062	0.187	0.312
	D1cc (Gy)	0.27	0–11.2	5.06	0.9–11.9	8.07	0.8–14.3	9.62	0.9–18.1	0.187	0.062	0.062
Spinal cord (*n* = 9)												
	Dmax (Gy)	0.01	0–8.5	10.0	3.6–16.7	9.8	4.5–13.0	11.41	5.8–17.8	**0.004**	**0.004**	**0.004**
Esophagus (*n* = 8)												
	Mean dose (Gy)	0	0–0.4	2.11	0.7–4.0	2.28	1.0–4.3	2.74	0.8–6.1	**0.008**	**0.008**	**0.014**
	Dmax (Gy)	0	0–11.1	8.60	4.1–13.7	7.97	6.4–15.6	11.85	5.8–17.4	**0.016**	**0.016**	**0.008**
Chest wall (*n* = 9)												
	Dmax (Gy)	44.35	31.0–75.9	39.72	28.9–73.0	41.68	29.1–76.3	44.01	34.4–72.8	0.734	0.652	0.203
	D30cc (Gy)	17.6	12.3–30.6	24.19	17.2–35.7	24.06	15.7–33.9	24.09	15.5–32.3	**0.019**	**0.019**	**0.019**
Skin (*n* = 8)												
	Dmax (Gy)	14.32	10.4–28.6	23.97	17.9–35.4	23.8	19.1–31.2	24.15	14.8–32.6	**0.008**	**0.008**	**0.008**

OARs, organs at risk; PBT, proton beam therapy; 3D-CRT, three-dimensional conformal radiotherapy; IMRT, intensity-modulated radiotherapy; VMAT, volumetric-modulated arc therapy, CGE, cobalt gray equivalent; fx, fractions; V5–40; percentage volume of tissue receiving 5–40 Gy; Dmax, maximum dose; D1cc, the dose delivered to 1 cubic centimeter volume; D30cc, the dose delivered to 30 cubic centimeter volume.

**Table 4:** Summary and comparison of the dosimetric parameters of tumors located close to the chest wall (60 CGE/4 fx = 2, 50 CGE/4 fx = 18, 70 CGE/10 fx = 1, and 60 CGE/10 fx = 1).

OARs		PBT		3D-CRT		IMRT		VMAT		*p*-Value		
		Median	Range	Median	Range	Median	Range	Median	Range	PBT vs.3D-CRT	PBT vs.IMRT	PBT vs.VMAT
Total lung (*n* = 22)												
	V5 (%)	9.61	3.6–14.7	14.32	6.0–21.6	14.12	6.0–20.8	12.49	4.8–20.3	**<0.001**	**<0.001**	**<0.001**
	V10 (%)	7.53	2.8–12.1	9.92	4.1–15.1	9.63	4.3–15.0	8.98	4.0–14.0	**<0.001**	**<0.001**	**<0.001**
	V15 (%)	6.02	2.2–10.0	6.94	2.7–11.6	6.86	2.9–11.5	6.13	3.0–10.6	**0.001**	**0.009**	0.222
	V20 (%)	4.91	1.7–8.5	4.99	2.1–9.0	4.88	2.1–8.9	4.37	2.1–8.1	0.085	0.849	**0.018**
	V30 (%)	3.12	1.2–5.7	2.75	1.2–5.6	2.67	0.9–5.2	2.52	1.0–4.7	**0.006**	**0.001**	**<0.001**
	V40 (%)	1.91	0.8–4.0	1.76	0.7–3.7	1.59	0.5–3.1	1.61	0.5–2.9	**<0.001**	**<0.001**	**<0.001**
	Mean dose (Gy)	2.55	0.9–4.2	3.54	1.7–5.8	3.47	1.8–5.5	3.4	1.7–5.3	**<0.001**	**<0.001**	**<0.001**
Heart (*n* = 22)												
	V5 (%)	0	0–1.7	2.75	0–59.5	4.45	0–62.26	5.19	0–56.0	**0.002**	**0.002**	**0.002**
	V10 (%)	0	0–1.2	0	0–19.9	0.04	0–21.9	0	0–18.9	**0.014**	**0.002**	**0.004**
	V15 (%)	0	0–0.8	0	0–5.1	0	0–5.8	0	0–5.7	0.181	0.181	0.059
	V20 (%)	0	0–0.6	0	0–1.2	0	0–1.5	0	0–1.2	1	0.371	1
	V30 (%)	0	0–0.3	0	0	0	0	0	0	1	1	1
	V40 (%)	0	0–0.1	0	0	0	0	0	0	1	1	1
	Mean dose (Gy)	0	0–0.4	1.14	0–6.7	1.18	0–7.1	1.28	0–6.5	**<0.001**	**<0.001**	**<0.001**
	Dmax (Gy)	0.02	0–63.74	8.43	0.4–24.9	10.68	0.4–26.7	9.25	0–26.1	**<0.001**	**<0.001**	**<0.001**
Proximal bronchial tree (*n* = 19)												
	Dmax (Gy)	3.69	0–38.4	9.35	0.5–34.0	10.8	0.5–33.6	10.36	0.5–36.2	0.104	**0.020**	**0.009**
	D1cc (Gy)	0.62	0–21.7	4.92	0.4–22.3	5.77	0.4–24.4	4.61	0.4–24.0	**<0.001**	**<0.001**	**<0.001**
Spinal cord (*n* = 22)												
	Dmax (Gy)	0	0–13.8	7.49	2.9–23.8	7.3	2.9–18.1	8.66	4.1–22.1	**<0.001**	**<0.001**	**<0.001**
Esophagus (*n* = 22)												
	Mean dose (Gy)	0	0–1.4	2.3	0.5–7.5	2.22	0.6–8.0	2.57	0.7–7.6	**<0.001**	**<0.001**	**<0.001**
	Dmax (Gy)	0.01	0–17.5	8.27	3.8–14.8	9.5	5.0–17.2	10.42	5.2–17.8	**<0.001**	**<0.001**	**<0.001**
Chest wall (*n* = 22)												
	Dmax (Gy)	55.69	35.3–74.9	60.48	34.9–91.2	60.59	34.5–77.1	61.29	36.4–77.0	**<0.001**	**0.001**	**<0.001**
	D30cc (Gy)	26.79	15.5–38.3	28.83	20.2–38.8	27.51	18.8–35.7	26.93	19.8–34.0	**0.008**	0.808	0.874
Skin (*n* = 21)												
	Dmax (Gy)	16.24	9.8–38.1	23.69	13.5–39.7	24.68	16.5–40.5	24.69	13.2–41.4	**<0.001**	**<0.001**	**<0.001**

OARs, organs at risk; PBT, proton beam therapy; 3D-CRT, three-dimensional conformal radiotherapy; IMRT, intensity-modulated radiotherapy; VMAT, volumetric-modulated arc therapy, CGE, cobalt gray equivalent; fx, fractions; V5–40; percentage volume of tissue receiving 5–40 Gy; Dmax, maximum dose; D1cc, the dose delivered to 1 cubic centimeter volume; D30cc, the dose delivered to 30 cubic centimeter volume.

## Data Availability

The data for this study, though not available in a public repository, will be made available to other researchers upon reasonable request to the corresponding author.

## Supplementary Materials

The following are available online at https://www.mdpi.com/article/10.3390/cancers13246356/s1, Figure S1: The volume–dose metrics comparisons for the lungs and heart according to the different modalities in all the cases (*n* = 42). Table S1: Summary and comparison of the dosimetric comparisons of all the cases (*n* = 42).
