# Supplementary material for: Dosimetric Comparisons between Proton Beam Therapy and Modern Photon Radiation Techniques for Stage I Non-Small Cell Lung Cancer According to Tumor Location
Source: Cancers (Basel). 2021 Dec 17;13(24):6356. doi: 10.3390/cancers13246356 (PMC8699272; doi:10.3390/cancers13246356)
Supplement: Supplementary file 1 [file cancers-13-06356-s001.zip › cancers-1509492-supplementary.pdf]

Supplementary Table S1. Summary and comparison of dosimetric comparison of all cases (n = 42)

PTV and OARs	PBT		3D-CRT		IMRT		VMAT		*p* value		
	**Median**	**Range**	**Median**	**Range**	**Median**	**Range**	**Median**	**Range**	**PBT vs. 3D-CRT**	**PBT vs. IMRT**	**PBT vs. VMAT**
PTV-SABR only (n = 33)											
CI	1.21	1.0–1.5	1.19	1.0–1.3	1.02	1.0–1.1	1.04	1.0–1.1	0.276	**<0.001**	**<0.001**
GI	4.49	3.6–6.7	4.88	4.2–5.6	4.42	3.7–5.7	4.24	3.7–5.2	0.113	0.201	**0.001**
Dmax (%)	116.72	107.0–134.2	122.18	116.7–133.1	130.58	122.0–137.2	130.4	123.1–136.0	**0.015**	**<0.001**	**<0.001**
Dmin (%)	89.65	69.4–99.5	86.4	77.2–91.8	93.35	83.0–96.8	92.93	87.2–95.9	0.053	**0.005**	**0.037**
PTV-Hypofractionated only (n = 9)											
CI	1.2	1.1–1.4	1.22	1.20–1.29	1.03	1.0–1.3	1.06	1.0–1.2	0.812	**0.016**	0.078
GI	4.48	3.4–6.1	5.03	4.1–5.7	5.16	4.9–6.9	4.64	3.7–5.4	0.641	0.055	0.742
Dmax (%)	115.5	101.0–123.2	120.41	116.5–131.0	109.99	107.4–112.5	112.63	107.6–115.4	**0.008**	0.203	0.496
Dmin (%)	86.77	51.1–96.8	86.77	59.6–89.6	93.33	75.2–95.2	90.03	76.1–98.5	0.912	0.074	**0.019**
Total lung (n = 42)											
V5 (%)	10.79	3.6–18.7	16.85	6.0–28.7	16.38	6.0–31.2	16.16	4.8–27.8	**<0.001**	**<0.001**	**<0.001**
V10 (%)	8.5	2.8–16.2	11.6	4.1–19.9	10.93	4.3–19.2	10.82	4.0–19.8	**<0.001**	**<0.001**	**<0.001**
V15 (%)	7.06	2.2–12.8	8.5	2.7–16.3	7.69	2.9–16.2	7.45	3.0–14.2	**<0.001**	**<0.001**	0.065
V20 (%)	5.85	1.7–10.9	5.98	2.1–12.4	5.47	2.1–12.7	5.14	2.1–12.3	**0.001**	0.864	**0.017**
V30 (%)	3.72	1.2–8.1	3.33	1.2–8.2	3.24	0.9–7.7	3.03	1.0–8.7	**0.027**	**0.001**	**<0.001**
V40 (%)	2.26	0.8–5.7	2.02	0.7–4.2	1.89	0.5–4.2	1.85	0.5–3.8	**<0.001**	**<0.001**	**<0.001**
Mean dose (Gy)	2.99	0.1–5.4	4.33	1.7–7.3	4.07	1.8–6.8	3.95	1.7–7.4	**<0.001**	**<0.001**	**<0.001**
Heart (n = 42)											
V5 (%)	0	0–5.3	4.37	0–70.5	7.02	0–72.8	8.64	0–64.0	**<0.001**	**<0.001**	**<0.001**
V10 (%)	0	0–4.0	0	0–39.3	0.22	0–51.4	0.45	0–41.5	**<0.001**	**<0.001**	**<0.001**

V15 (%)	0	0–3.1	0	0–25.1	0	0–29.4	0	0–20.5	**0.004**	**0.003**	**<0.001**
V20 (%)	0	0–2.0	0	0–10.2	0	0–14.3	0	0–8.7	**0.013**	**0.008**	**0.009**
V30 (%)	0	0–1.2	0	0–1.9	0	0–3.8	0	0–2.1	0.675	0.554	1
V40 (%)	0	0–0.5	0	0–0.3	0	0–1.0	0	0–0.6	0.789	1	1
Mean dose (Gy)	0	0–1.1	1.42	0–9.7	1.57	0–11.2	1.70	0–9.2	**<0.001**	**<0.001**	**<0.001**
Dmax (Gy)	0.18	0–63.7	9.96	0.4–54.8	11.73	0.4–63.8	12.96	0–64.4	**<0.001**	**<0.001**	**<0.001**
Proximal bronchial tree (n = 33)											
Dmax (Gy)	4.02	0–69.0	14.33	0.5–77.5	13.12	0.5–69.2	13.09	0.5–71.4	**<0.001**	**0.001**	**0.001**
D1cc (Gy)	0.78	0–65.3	8.08	0.4–61.7	8.27	0.4–60.5	9.19	0.4–62.5	**<0.001**	**<0.001**	**<0.001**
Great vessel (n = 17)											
Dmax (Gy)	57.59	3.5–79.6	50.53	11.9–81.4	53.08	13.7–74.5	53.03	17.2–75.2	1	0.517	0.698
Hilar major vessels (n = 25)											
Dmax (Gy)	52.17	0–78.3	51.5	0.6–81.7	53.31	0.6–77.5	55.62	0.5–78.8	0.916	0.182	0.300
D1cc (Gy)	26.81	0–66.6	25.12	0.4–70.9	25.82	0.4–63.3	28.16	0.3–64.7	0.691	0.508	0.381
Spinal cord (n = 42)											
Dmax (Gy)	0.01	0–14.1	8.23	2.9–23.8	8.85	2.9–20.2	9.5	4.1–29.6	**<0.001**	**<0.001**	**<0.001**
Esophagus (n = 41)											
Mean dose (Gy)	0	0–1.4	2.33	0.5–16.0	2.29	0.6–16.9	2.72	0.7–17.6	**<0.001**	**<0.001**	**<0.001**
Dmax (Gy)	0.08	0–17.5	9.26	3.8–29.8	11.37	5.0–36.9	11.76	5.2–26.8	**<0.001**	**<0.001**	**<0.001**
Chest wall (n = 38)											
Dmax (Gy)	55.69	31.0–75.9	60.31	28.9–91.2	60.24	29.1–77.1	61.15	34.4–77.0	**<0.001**	**0.005**	**<0.001**
D30cc (Gy)	26.03	12.3–44.0	27.13	17.2–39.6	27.39	15.7–41.6	26.76	15.5–39.7	**<0.001**	**0.017**	0.072
Skin (n = 38)											
Dmax (Gy)	17.23	9.8–54.8	24.28	13.5–39.7	24.89	16.5–40.6	24.74	13.2–41.4	**<0.001**	**<0.001**	**<0.001**

OARs, organs at risk; PBT, proton beam therapy; 3D-CRT, three-dimensional conformal radiotherapy; IMRT, intensity-modulated radiotherapy; VMAT, volumetric modulated arc therapy, CGE, Cobalt Gray Equivalent; fx, fractions; PTV, planning target volume;

SABR, stereotactic ablative radiotherapy; CI, conformity index; GI, gradient index; Dmax, maximum dose; Dmin, minimum dose; V5-40; percentage volume of tissue receiving 5-40 Gy; D1cc, the dose delivered to 1 cubic centimeter volume; D30cc, the dose delivered to 30 cubic centimeter volu

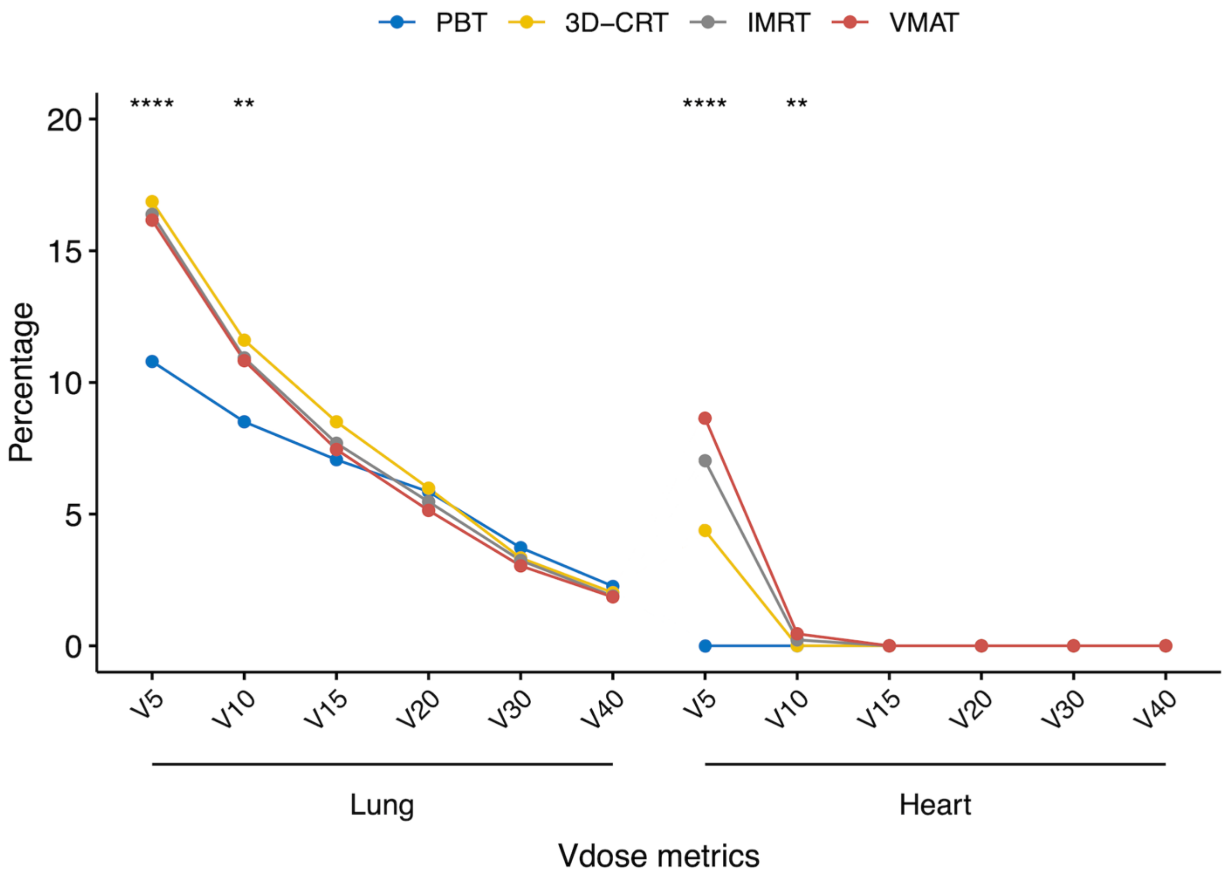

Supplementary Figure S1. The volume-dose metrics comparisons for the lung and heart according to different modalities in all cases (n = 42). The points are representing the median values of respective parameters. The Kruskal-Wallis test was used to determine the significant difference among the groups.

Not significant are not shown. * ≤ 0.05; ** ≤ 0.01; *** ≤ 0.001; **** ≤ 0.0001.

PBT, proton beam therapy; 3D-CRT, three-dimensional conformal radiotherapy; IMRT, intensity-modulated radiotherapy; VMRT, volumetric-modulated arc therapy; Vdose, volume dose; V5-40, percentage volume of tissue receiving 5-40 Gy.
